# Structure-function correlations in Retinitis Pigmentosa patients with partially preserved vision: a voxel-based morphometry study

**DOI:** 10.1038/s41598-017-11317-7

**Published:** 2017-09-12

**Authors:** Ana Rita Machado, Andreia Carvalho Pereira, Fábio Ferreira, Sónia Ferreira, Bruno Quendera, Eduardo Silva, Miguel Castelo-Branco

**Affiliations:** 1 Faculty of Medicine, University of Coimbra, Coimbra, Portugal; 2 Institute for Biomedical Imaging and Life Sciences (CNC.IBILI), Faculty of Medicine, University of Coimbra, Coimbra, Portugal; 3 Sackler Institute for Translational Neurodevelopment, Forensic and Neurodevelopmental Sciences Department, Institute of Psychiatry, Psychology and Neuroscience, King’s College, London, United Kingdom; 4 CiBIT, Institute of Nuclear Sciences Applied to Health (ICNAS P), Brain Imaging Network of Portugal, University of Coimbra, Coimbra, Portugal

## Abstract

Retinitis Pigmentosa is a group of hereditary retinal dystrophy disorders associated with progressive peripheral visual field loss. The impact of this retinal loss in cortical gray matter volume has not been addressed before in Retinitis Pigmentosa patients with low vision. Voxel-based morphometry was applied to study whole brain gray matter volume changes in 27 Retinitis Pigmentosa patients with partially preserved vision and 38 age- and gender-matched normally sighted controls to determine whether peripheral visual loss can lead to changes in gray matter volume. We found significant reductions in gray matter volume that were restricted to the occipital cortex of patients. The anteromedial pattern of reduced gray matter volume in visual primary and association cortices was significantly correlated with the extent of the peripheral visual field deficit in this cohort. Moreover, this pattern was found to be associated with the extent of visual field loss. In summary, we found specific visual cortical gray matter loss in Retinitis Pigmentosa patients associated with their visual function profile. The spatial pattern of gray matter loss is consistent with disuse-driven neuronal atrophy which may have clinical implications for disease management, including prosthetic restoration strategies.

## Introduction

Retinitis Pigmentosa (RP) is an important cause of human visual disability characterized by early peripheral visual field defects (VFD). The non-syndromic form of RP has a worldwide prevalence of about 1/4000 individuals^1–4^. It is classified as an inherited retinal dystrophy disorder which is characterized by photoreceptor degeneration and subsequent visual loss^3^. In the typical form of RP, retinal degeneration affects rod photoreceptors in the mid-periphery, advancing towards the macula and fovea with cone photoreceptors affected only in later stages. Patients initially present with nyctalopia, followed by the progressive peripheral VFD, and eventually blindness. The age of onset is highly variable, ranging from childhood to mid-adulthood^1, 2^.

Voxel-based morphometry (VBM) is a well-established technique^5–7^ that has been used to report local gray matter (GM) volume alterations in several disorders leading to VFD^8–19^. However, some of these studies have only focused on patients whose late VFD had already evolved to blindness and include multiple neuro-ophthalmological pathologies within the same study. Thus, different mechanisms of visual loss and putative brain adaptation were likely present^16–18^. VBM studies in patients with partially preserved vision due to VFD have mainly concerned glaucoma, also characterized by peripheral VFD, or age-related macular degeneration (AMD) characterized by central or pericentral VFD^8–15^. GM loss has been reported in the visual cortex of AMD and glaucoma patients in cortical representations matching the retinal lesion projection zones^8^. Several other studies have found cortical changes, mainly volumetric reduction, in patients with central retinal degeneration^10, 11, 19, 20^. Furthermore, in a morphometric study involving patients with hereditary macular dystrophies with central scotomata, the authors found a correlation between scotomata size and GM volume, suggesting a direct association between the lack of visual input and GM atrophy^11^. Regarding peripheral VFD, the focus of morphometry studies has been mainly on glaucoma^8, 12–15, 21^. Li *et al.* (2012)^12^ had analyzed the extraoccipital cortex in progressive stages of primary open-angle glaucoma and did not find GM volumetric changes in the early stage group. On the contrary, in the advanced-late stage group, reduced GM volume was found in multiple cortical brain regions including the primary visual cortex^12^. Conversely, these authors found higher GM volume in regions neighboring the most damaged brain region, which may reflect the possibility of structural reorganization. Similar studies also reported volumetric decreases and increases in several brain regions in glaucoma^14, 15^. To the best of our knowledge, only one study has investigated the presence of visual cortical volumetric reduction due to peripheral VFD in disorders other than glaucoma. In that study, wider calcarine fissures were reported in nine patients with retinal pathologies, particularly in the anterior and middle portions, which is also consistent with peripheral VFD^22^. Nevertheless, only four patients presented with pigmentary degeneration of the retina and, since the authors reported only the calcarine fissure width, it remains unclear whether more widespread changes related to the visual loss were present.

The present work aims to determine whether progressive peripheral vision loss in RP patients might lead to structural cortical changes, not only in the visual cortex but also in the whole brain. We hypothesize that RP patients with low vision would have reduced GM volume, mainly in the visual cortex due to visual field constriction, as previously shown for other late-onset VFD pathologies^8, 11, 14^. Furthermore, considering the VFD similarities with glaucoma, neighboring regions with increased GM volume might also be present^14, 15^. This question is relevant as GM alterations caused by peripheral VDF in a pathology other than glaucoma have not been previously addressed. For that purpose, a whole-brain VBM analysis was used to investigate the possible link between peripheral VDF and GM abnormalities in the currently largest cohort of low vision RP patients included in morphometric studies.

## Results

### Demographic and visual data

Table 1 shows the mean or median values for the RP and control groups for demographic and visual data. RP patients and controls were age- [*U* = 462.50, *p* = 0.501], gender- [χ^2^_(1)_ = 0.05, *p* = 0.508], and handedness-matched [χ^2^_(2)_ = 2.96, *p* = 0.228; 2 RP patients with missing data]. Visual acuity [right eye *U* = 1026.00, *p* = 4.018 × 10^−12^ and left eye *U* = 1004.00, *p* = 1.838 × 10^−11^] and retinal thickness [right eye *t*_(29.76)_ = −8.07, *p* = 5.535 × 10^−9^ and left eye *t*_(30.49)_ = −8.48, *p* = 1.615 × 10^−9^; 1 RP patient with missing data] of both eyes were significantly reduced for the RP group as compared to the control group. No significant statistical differences were observed for RNFL thickness of both eyes between RP patients and controls [right eye *t*_(30.84)_ = 0.01, *p* = 0.994 and left eye *t*_(32.68)_ = −0.21, *p* = 0.831; 1 RP patient with missing data]. It is worth mentioning that 4 patients presented with retinal edema (subjects 10, 13, 21, and 26 in Table 2), which may lead to an overestimation of the retinal and the RNFL thickness. The mean deficit of the visual field was significantly higher for the RP group when compared to the control group in both eyes [right eye *U* = 0.50, *p* = 7.274 × 10^−11^ and left eye *U* = 4.00, *p* = 1.746 × 10^−10^; 4 RP patients could not perform the examination with both eyes and 1 RP patient with the left eye]. Patients’ visual field diameter ranged from 5 to 48 degree in the right eye and 10 to 48 degrees in the left eye, as assessed with static perimetry. It was not possible to estimate the visual field extent of 4 RP patients, who could not perform the exam with both eyes, and from the left eye of 1 RP patient. Additionally, no statistical differences were found between the right and left eye within the RP and control groups for any of the visual parameters studied: visual acuity [RP *Z* = −0.11, *p* = 0.911; controls *Z* = −0.57, *p* = 0.566], retinal thickness [RP *t*_(25)_ = 0.60, *p* = 0.551; controls *t*_(37)_ = 1.34, *p* = 0.189], RNFL thickness [RP *t*_(25)_ = 0.71, *p* = 0.483; controls *t*_(37)_ = 1.34, *p* = 0.189], visual field mean deficit [RP *Z* = 1.01, *p* = 0.314; controls *Z* = −0.26, *p* = 0.793], and visual field diameter [RP *Z* = 1.41, *p* = 0.157; controls had the maximum measured visual field diameter of 48 degrees, thus, a constant value]. Lastly, no correlation was present either between disease duration and visual field mean deficit (Spearman *rho* = 0.150, *p* = 0.506) or between disease duration and visual field diameter (Spearman *rho* = 0.074, *p* = 0.743) in the RP group, which might be explained by the clinical difficulty in correctly establishing true disease onset which has a long preclinical phase.

**Table 1:** Demographic and visual data comparisons between groups. *p* < 0.05.

Characteristic	Eye	RP (n = 27)	Control (n = 38)	Test value, *p-*value
Age (years)		41.00 (21.00)	39.00 (18.00)	*U* = 462.50, *p* = 0.501
Gender (female/male)		12/15	18/20	χ^2^_(1)_ = 0.05, *p* = 0.508
Handedness (right/left)^*^		24/1	36/2	χ^2^_(2)_ = 2.96, *p* = 0.228
Visual acuity (logMAR)	Right	−0.40 (0.26)	0.00 (0.12)	*U* = 1026.00, *p* = 4.018 × 10^−12^
	Left	−0.40 (0.30)	0.00 (0.11)	*U* = 1004.00, *p* = 1.838 × 10^−11^
Retinal thickness (µm)^†^	Right	234.23 ± 32.12	287.42 ± 11.93	*t*_(29.76)_ = −8.07, *p* = 5.535 × 10^−9^
	Left	233.23 ± 30.75	287.08 ± 12.26	*t*_(30.49)_ = −8.48, *p* = 1.615 × 10^−9^
RNFL thickness (µm)^†^	Right	95.38 ± 25.17	95.34 ± 10.35	*t*_(30.84)_ = 0.01, *p* = 0.994
	Left	93.65 ± 19.86	94.55 ± 9.37	*t*_(32.68)_ = −0.21, *p* = 0.831
Visual field mean deficit (dB)^‡^	Right	20.00 (7.40)	0.30 (0.30)	*U* = 0.50, *p* = 7.274 × 10^−11^
	Left	19.90 (6.10)	0.30 (0.22)	*U* = 4.00, *p* = 1.746 × 10^−10^

RP = Retinitis Pigmentosa; n = number of participants; RNFL = retinal nerve fiber layer; ^*^Two patients with missing data; ^†^One patient with missing data; ^‡^Four patients could not perform the exam with both eyes and one with the left eye. Values are mean ± standard deviation or median (interquartile range). Significance level at *p* < 0.05.

**Table 2:** Demographic data and visual characterization of the Retinitis Pigmentosa patients.

Subject	Age (years)	Gender	Handedness	Onset age (years)	Disease duration (years)	Visual acuity (decimals/logMAR)		Retinal thickness (µm)		RNFL thickness (µm)		Visual field diameter (degrees)		Visual field mean deficit (dB)	
						RE	LE	RE	LE	RE	**LE**	**RE**	**LE**	**RE**	**LE**
1	42	M	R	18	24	0.50/−0.30	0.60/−0.22	200	194	116	100	5	10	23.20	22.40
2	34	F	R	3	31	0.60/−0.22	0.30/−0.52	265	281	101	101	20	20	20.10	20.30
3	58	M	a	14	44	0.05/−1.30	0.05/−1.30	206	209	54	53	^†^	^†^	^†^	^†^
4	38	F	R	6	32	0.28/−0.55	0.80/−0.10	221	216	109	133	25	25	16.89	18.10
5	45	F	R	39	6	0.66/−0.18	0.80/−0.10	184	192	81	76	10	10	14.90	15.40
6	47	F	R	14	33	0.40/−0.40	0.33/−0.48	250	239	107	88	5	^†^	22.30	^†^
7	35	M	R	6	29	0.50/−0.30	0.90/−0.05	243	226	91	89	10	10	22.00	21.90
8	41	M	R	11	30	0.66/−0.18	0.50/−0.30	209	218	75	69	10	10	23.00	22.70
9	29	M	R	6	23	0.40/−0.40	0.40/−0.40	185	189	93	92	10	10	22.80	23.10
10	50	M	L	8	42	0.40/−0.40	0.66/−0.18	207	205	73	79	25	25	17.39	17.70
11	63	M	R	45	18	0.40/−0.40	0.12/−0.92	219	223	73	82	10	10	18.60	18.89
12	50	M	R	16	34	0.50/−0.30	0.50/−0.30	203	201	62	70	10	10	20.00	20.20
13	61	M	*	10	51	0.60/−0.22	0.30/−0.52	274	268	54	70	10‡	10‡	30.09‡	29.89‡
14	20	M	R	6	14	0.66/−0.22	0.66/−0.22	228	225	99	106	15	20	21.20	20.50
15	50	F	R	29	21	0.50/−0.30	0.40/−0.40	222	224	100	95	10	10	12.70	16.30
16	63	F	R	10	53	0.12/−0.92	0.20/−0.70	236	242	79	86	^†^	^†^	^†^	^†^
17	66	F	R	18	48	0.28/−0.55	0.12/−0.92	291	295	90	91	48	48	4.40	5.20
18	52	M	R	7	45	0.05/−1.30	0.05/−1.30	227	215	101	95	^†^	^†^	^†^	^†^
19	28	F	R	15	13	0.60/−0.22	0.50/−0.30	198	197	98	95	40	40	14.50	13.90
20	23	M	R	16	7	0.66/−0.18	0.50/−0.30	266	254	143	130	10	10	21.80	22.10
21	25	M	R	14	11	0.50/−0.30	0.40/−0.40	245	242	95	101	40	40	11.00	11.50
22	66	F	R	27	39	0.50/−0.30	0.50/−0.30	248	254	108	95	10	10	19.50	19.60
23	23	F	R	1	22	0.33/−0.48	0.20/−0.70	*	*	*	*	20	20	21.40	21.30
24	36	M	R	26	10	0.20/−0.70	0.33/−0.48	308	291	166	127	^†^	^†^	^†^	^†^
25	38	F	R	32	6	0.40/−0.40	1.00/0.000	258	249	128	128	20	20	16.80	17.39
26	32	M	R	12	20	0.40/−0.40	0.40/−0.40	229	240	93	102	10	10	22.80	22.60
27	31	M	R	1	30	0.40/−0.40	0.80/−0.10	268	273	91	92	48	48	1.70	1.00

M = male; F = female; L = left; R = right; LE = left eye; RE = right eye; RNFL = retinal nerve fiber layer; *Data not available; ^†^Patient could not perform the exam; ^‡^Patient evaluated with Humphrey Field Analyzer.

### VBM data

**Figure 1:**
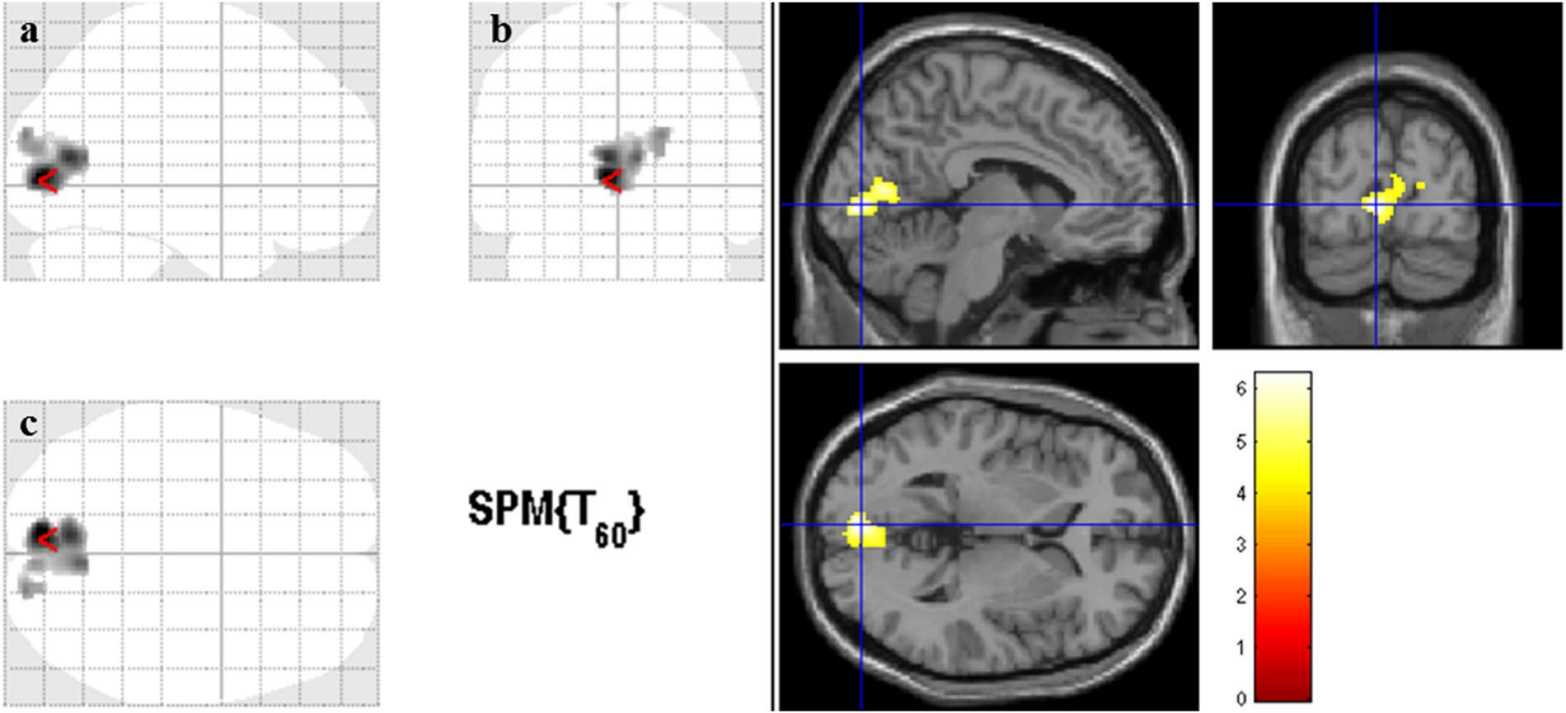
Gray matter volume reduction in Retinitis Pigmentosa patients. Cortical areas with significant gray matter volume reduction in Retinitis Pigmentosa patients when compared to controls for the whole brain analysis (*p* < 0.01, False-Discovery-Rate correction for multiple comparisons and a cluster size correction of 20 voxels). The color bar indicates the range of *t*-values with white/yellow representing more significant differences (higher *t*-values), orange indicating less significant differences (middle range *t*-values), and red indicating nonsignificant differences (lower *t*-values). (**a**) Sagittal view; (**b**) Coronal view; (**c**) Axial view. The cursor is positioned in the Montreal Neurological Institute coordinates with the maximum *t*-value [−6, −84, 2 mm (red arrow)].

Figure 1 shows the statistical parametric map of reduced GM volume in RP patients when compared to controls. Reduced GM volume was found in both hemispheres and involved the following brain regions: right and left calcarine, right and left lingual, right and left cuneus, and right occipital superior gyrus (Table 3). The analysis with the contrast RP > Controls yielded no statistically significant results (i.e. no regions with significant increased GM volume was found in RP patients).

**Table 3:** Voxel-Based morphometry results for group comparisons showing regions of gray matter volumetric reduction in the Retinitis Pigmentosa group.

Cluster	Number of voxels	Peak voxel t-value, *p*-value	Peak voxel MNI coordinates x, y, z (mm)	Cortical areas
Cluster 1	890	6.30, <0.0001	−6, −84, 2	Right and left calcarine; right and left lingual; right and left cuneus; right occipital superior gyrus

MNI = Montreal Neurological Institute coordinate system.

**Figure 2:**
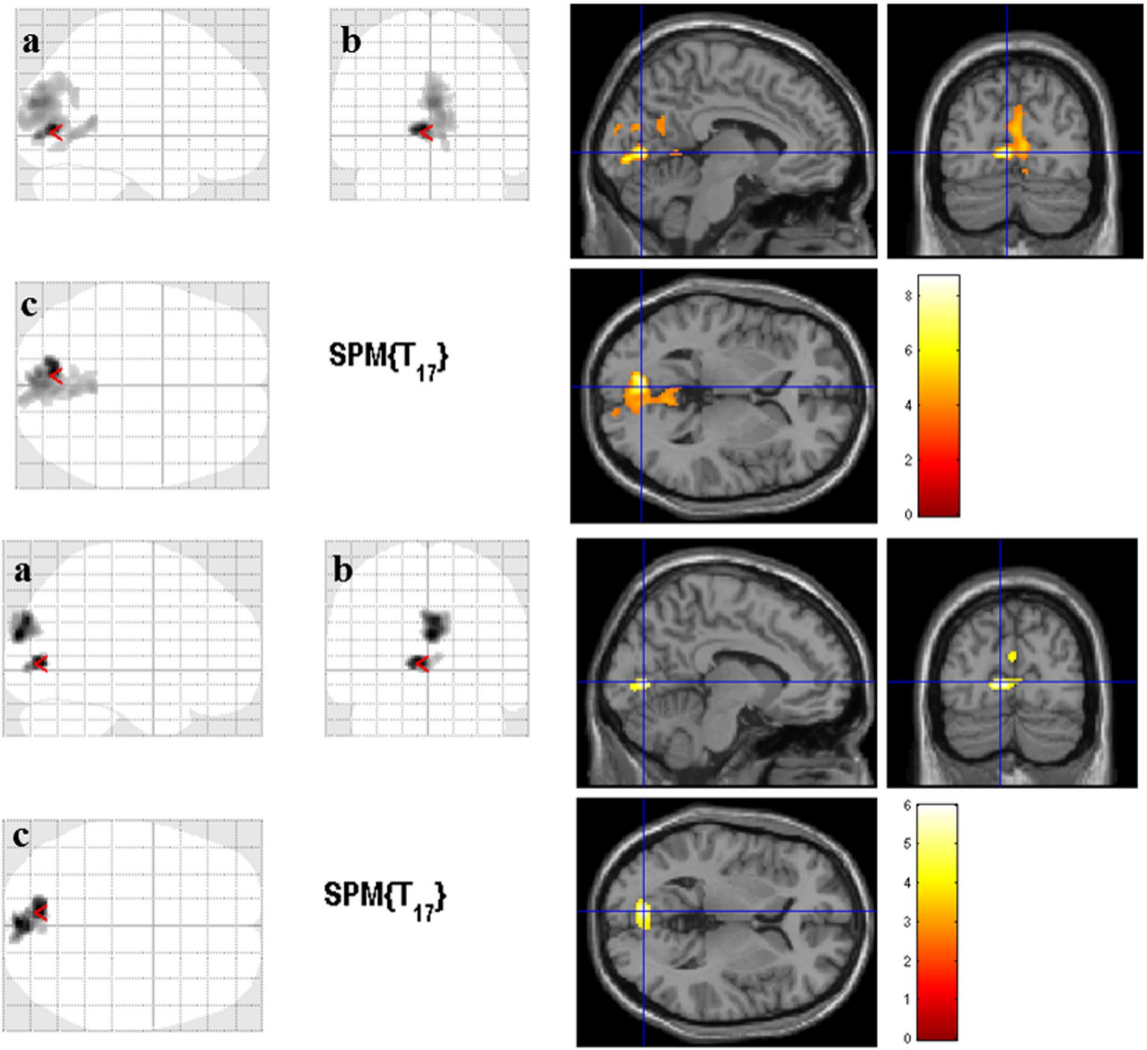
Correlations between cortical gray matter volume and visual field loss in the Retinitis Pigmentosa patients. The same altered regions (Fig. 1) present a correlation between cortical gray matter volume and visual field extent in the Retinitis Pigmentosa patients (*p* < 0.001 with a cluster correction of 150 voxels). In the upper part, it is represented the direct (positive) correlation between gray matter volume and visual field diameter (in degrees). A smaller visual field diameter corresponds to reduced gray matter volume. In the lower part, it is displayed the inverse (negative) correlation between mean deficit (in dB) and gray matter volume. High mean deficit values correspond to reduced gray matter volumes. The color bar indicates the range of *t*-values with white/yellow representing more significant differences (higher *t*-values), orange indicating less significant differences (middle range *t*-values), and red indicating nonsignificant differences (lower *t*-values). (**a**) Sagittal view; (**b**) Coronal view; (**c**) Axial view. The cursor is positioned in the Montreal Neurological Institute coordinates with the maximum *t*-value [upper part −6, −76, 2 mm; lower part −8, −78, 4 mm (red arrow)].

Figure 2 shows the statistical parametric maps of statistically significant correlations between cortical GM volume and visual field loss (visual field diameter and mean deficit variables) in RP patients. The same regions showed GM loss related with visual function deterioration. Correlation analysis between GM volume and preserved visual field diameter showed significant positive associations in the following brain regions: right and left cuneus, right and left calcarine, right and left lingual, right and left precuneus. Correlation analysis between visual field mean deficit and GM volume showed negative associations in the following brain regions: right and left calcarine and right and left lingual, and in right and left cuneus and right occipital superior, respectively (Table 4). No other visual parameters (visual acuity, retinal thickness, or RNFL thickness) or disease duration showed statistically significant correlations with GM volume.

**Table 4:** Clusters showing significant correlations between cortical gray matter volume and visual field loss severity in the Retinitis Pigmentosa group.

Visual Parameter	Cluster	Number of voxels	Peak voxel t -value, *p*-value	Peak voxel MNI coordinates x, y, z (mm)	Cortical areas
Visual field’s diameter (degrees)	Cluster 1	1625	8.70, <0.00001	−6, −76, 2	Right and left cuneus; Right and left calcarine; Right and left lingual; Right and left precuneus
Visual field’s mean deficit (dB)	Cluster 1	185	5.99, <0.00001	−8, −78, 4	Right and left calcarine; Right and left lingual
	Cluster 2	340	5.90, <0.00001	0, −92, 20	Right and left cuneus; Right occipital superior gyrus

MNI = Montreal Neurological Institute coordinate system.

## Discussion

Our results showed that Retinitis Pigmentosa (RP) patients with low (partially preserved) vision have significantly reduced gray matter (GM) volume when compared to healthy controls in several components of the primary and association visual cortices. Moreover, visual cortical atrophy in RP patients was associated with the magnitude of peripheral visual degeneration. To our knowledge, this is the first whole-brain morphometric study conducted in this pathology.

In our study, GM loss was found bilaterally in calcarine sulci, lingual gyri, cuneus, and also in the right occipital superior gyrus. Patterns of loss in anteromedial occipital cortex were correlated with the extent of field loss (Fig. 2). Our findings are in line with previous studies showing that GM volume changes occur in the visual cortex of patients with visual field deficits (VFD) acquired late in life^8, 10–15, 19–23^.

Given that the visual cortex is organized into retinotopic maps, it is relevant to discuss whether our findings are consistent with the topology of visual loss. In the retina, each visual field representation has receptive fields at nearby locations in the image. Thus, these topological maps preserve the spatial arrangement of the retinal image especially in the primary visual cortex (V1), where adjacent stimuli in the visual field are represented in adjacent positions in the visual cortex. If progressive VFD occur over the years, the corresponding part of the visual cortex gradually loses its input. Thus, continued visual input loss due to VFD can result in structural changes at the visual cortex level, particularly in V1^24–26^. In this way, the typical peripheral VFD of RP corresponds to a partial loss of input to the more anterior and middle visual cortex, which is consistent with our findings. Accordingly, our data (group comparisons and correlation analyses with visual function parameters) corroborates this hypothesis since the observed GM reductions were present in these regions of the occipital cortex, while the more posterior regions were spared (Fig. 1). Moreover, this spatial pattern of reduced GM volume is consistent with previous VBM studies focusing in peripheral VFD^8, 22^ and, at the same time, mirror the GM volume reductions observed in the case of central VFD. In our study, brain areas showing GM volume reduction included the primary visual cortex but also extended towards the association cortices. These results emphasize that even in low vision patients, widespread GM atrophy is present in several regions of the primary and secondary visual cortices and beyond. Possible explanations for the observed GM reduction in the visual cortex are disuse-driven neuronal atrophy and/or transneuronal degeneration^23^. The structural alterations of afferent visual pathways in RP have been investigated by measuring retinal nerve fiber layer (RNFL) and retinal thickness, using optical coherence tomography (OCT). Retinal thinning in RP is consensual across studies^27–29^, and our findings of reduced retinal thickness as measured by OCT (Table 1) support this notion. On the other hand, RNFL findings have been contradictory in RP. It has been reported to be increased, decreased, or maintained within normal limits in several studies^29–31^. In the current work, the average RNFL thickness of RP patients is similar to the control group (Table 1), which suggests axonal integrity of the optic nerve. Optic pathway trans-synaptic neurodegeneration (Wallerian degeneration) is well established in patients with glaucoma-induced retinal ganglion cells (RGC) damage^9, 13–15, 23, 32^. In macular degeneration, volumetric loss of the visual pathway has also been observed^13, 32, 33^. In this case, the adverse effect of photoreceptor degeneration (first-order neurons) on RGC (third-order neurons) was proposed as a possible explanation in that disease^19^. Nevertheless, a diffusion tensor imaging study of the optic nerve in RP revealed pathologic structural alterations of the optic nerve^34^, while another found decreased integrity of the optic radiation in RP patients^35^. Further studies are necessary to assess a better understanding of the role of RGC and the visual pathway alterations in RP.

Contrarily to some studies in glaucoma^12, 14, 15^, we did not observe GM hypertrophy in RP patients when compared to controls. Our results of reduced GM volume restricted to the occipital region in the absence of GM increases suggests that, notwithstanding the VFD similarities, the neuropathological features may differ between the two conditions. In fact, the primary retinal damage differs between glaucoma and RP (i.e. RGC versus photoreceptors, respectively) which may lead to a distinct impact in the disease progression. However, we cannot rule out the possibility that the conservative whole-brain analysis used in this study might have missed subtle increases in GM volume.

In the correlation analysis for the RP group, GM volume was directly (positively) correlated with visual field diameter in an occipital cluster extending to the posterior region of the medial parietal cortex, and was inversely (negatively) correlated with the visual field mean deficit in two occipital clusters (Table 4). The cortical regions which correlate with visual parameters remarkably overlap with the regions of reduced GM volume observed in the between-group comparisons. No other visual parameters (visual acuity, retinal thickness, and RNFL thickness) or disease duration showed significant correlations with GM volume. One previous study showed that this measure of disease severity did not correlate with GM reductions caused by central VFD^11^. However, this was expected because the age of symptoms onset was reported by the patients which makes this parameter highly subjective and an imprecise measure of disease severity^1, 2, 36^. Thus, RP’s visual field loss, here characterized by the parameters visual field diameter and mean deficit, seems to be the only measure related to the GM volume atrophy. In other words, our data suggest that GM atrophy in the visual cortex of RP patients with partially preserved vision is likely driven by the extent of the visual field loss. In a recent functional magnetic resonance imaging (MRI) work from our group, which included retinotopic assessment of half of the RP patients from the current study, with more preserved visual function, V1 functional alterations were also dependent on the extent of visual field loss and not related to disease duration^36^. Our findings are in line with studies in other hereditary retinal dystrophies, which also reported correlations between the visual field loss and GM atrophy^11, 20^.

Our results support the hypothesis that atrophy and degeneration of the visual cortex can occur in patients with low vision even before blindness is settled. These findings may have important clinical implications in disease management because neurodegenerative pathophysiology may be early present in the course of the disease. Current investigational treatment modalities, such as gene replacement therapy, retinal or stem cell transplantation, pharmacologic neurotrophic factors, and neuroprosthetic devices should be implemented early in the course of the disease to avoid the progression to visual cortical atrophy^1, 2, 37^. Moreover, the success of retinal functional restoration highly depends on the integrity of the visual pathways to correctly lead the input to the visual cortex^38^. MRI has been proposed as a tool to evaluate to what extent an intervention may be effective^39^ since patients with severe cortical structural atrophy may not benefit from retinal input restoration^40^. Also, MRI studies allow brain structural and functional assessment after retinal implants, enabling pre- and post-intervention disease management^41^. Our results further support this possibility for RP patients with low vision.

In summary, we showed striate and extrastriate visual cortical GM reduction in RP patients with low vision, associated with the extent of visual field loss.

## Methods

### Subjects

Twenty-eight Retinitis Pigmentosa (RP) patients were recruited from the Ophthalmology Department at the University of Coimbra Hospital, Portugal. Forty-three age- and gender-matched controls were recruited from the volunteers’ database of the Institute for Biomedical Imaging and Life Sciences (IBILI) of the Faculty of Medicine, University of Coimbra, Portugal. All participants underwent an ophthalmologic examination (visual acuity assessment, automated perimetry, and optical coherence tomography [OCT]) and a structural magnetic resonance imaging (MRI) acquisition.

Patients with other neuro-ophthalmological or organic brain disorder were excluded from the study. One patient could not perform the MRI scan due to claustrophobia, being consequently excluded. Twenty-seven RP patients (12 females, 15 males; age range 20–66 years) were finally included (Table 2).

None of the controls participants had a history of neurological or organic brain disorders. Exclusion criteria were: alterations in the ophthalmologic examination (automated perimetry and OCT), eye disorders known a priori, and alterations in the MRI structural images. Controls were required to have normal or corrected to normal vision (visual acuity ranged from 0.80 to 1.33 decimals [−0.10 to 0.12 logMAR]). Four control subjects were excluded due to alterations on the ophthalmological examination and another participant was excluded due to excessive movement in the MRI acquisition. Thirty-eight healthy controls (18 females, 20 males; age range 22–74 years) were included.

The study was conducted in accordance with the Declaration of Helsinki and was approved by the Ethics Commission of Faculty of Medicine of University of Coimbra, Portugal. Written informed consent was obtained from all participants.

### Ophthalmic examination

All participants underwent an ophthalmological examination including visual acuity assessment, static visual field evaluation with automated perimetry, and macular and retinal nerve fiber layer (RNFL) thickness measurements with OCT. All assessments were performed in each eye separately. Ophthalmological examinations were evaluated by an experienced ophthalmologist (ES) to rule out eye disorders in the control group.

Visual acuity was determined for each participant using a decimal chart (VA-Snellen chart) and the values were converted to the logarithm of Minimum Angle of Resolution (logMAR) scale before performing the statistical analysis. The right eye and left eye were evaluated separately with the same chart. When patients’ visual acuity was so low that they could not read any of the largest letters or even count fingers, visual capacity was assessed with hand motion visual or light perception testing. The values for visual acuity in these patients (subjects 3 and 18 in Table 2) were considered to be 0.05 decimals (−1.30 logMAR).

**Figure 3:**
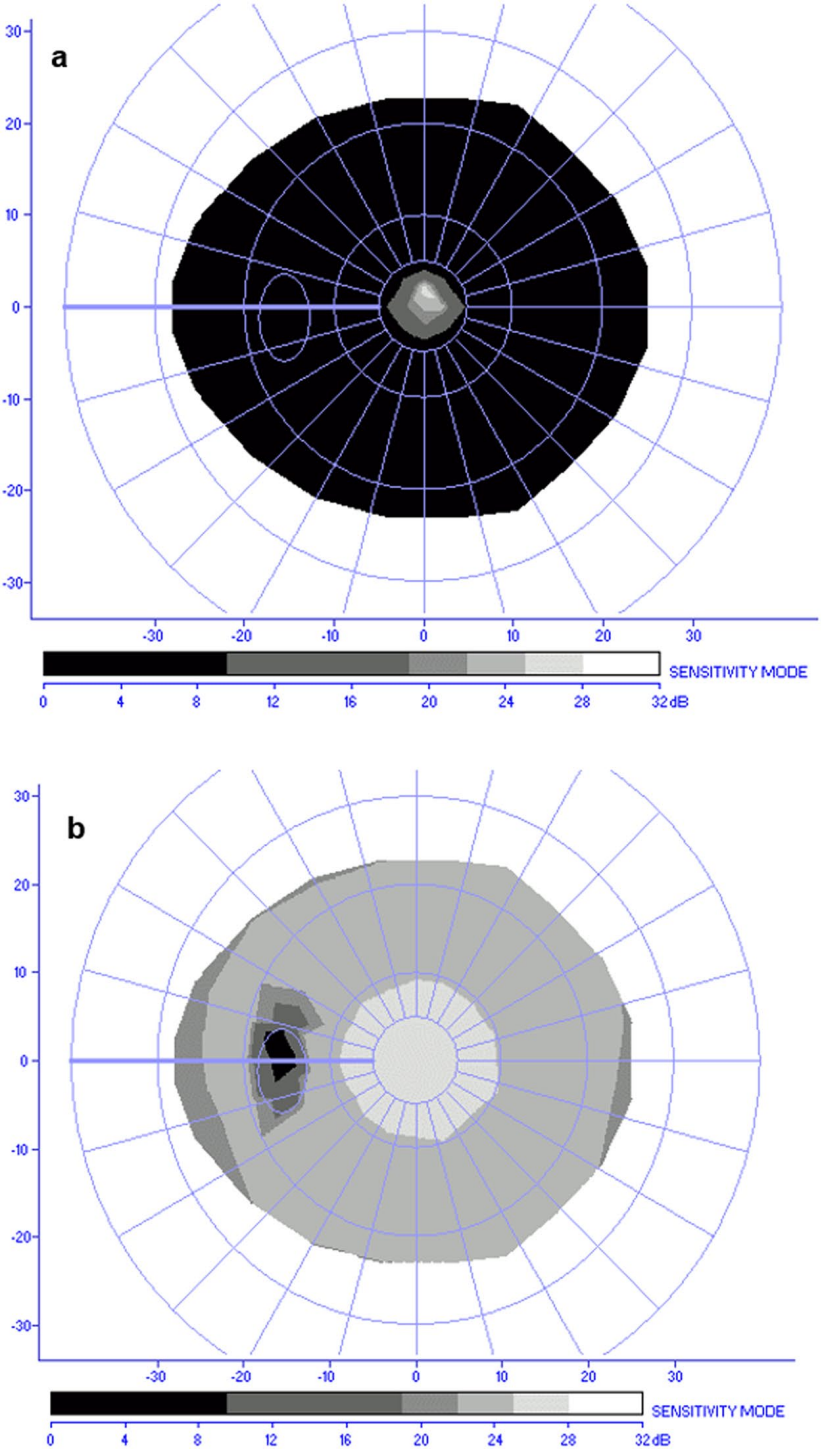
Static perimetry sensitivity maps. Examples of static perimetry sensitivity maps using the MonCv3 multifunction perimeter (Metrovision, France). (**a**) Visual field result of the left eye of a Retinitis Pigmentosa patient (subject 1 in Table 2 with a visual field diameter of about 10 degrees of visual angle). (**b**) Visual field result of the left eye of a control participant (48 degrees of visual angle in diameter). The colored bar represents the sensitivity values and the axes show the visual angle in degrees.

Static visual fields were evaluated using a MonCv3 multifunction perimeter (Metrovision, France) with a standardized program (except for 4 patients, who could not perform the exam for both eyes – subjects 3, 16, 18, and 24 in Tables 2 and 1 patient, who was unable to perform the exam using the left eye – subject 6 in Table 2). These RP patients could not perform the examination due to low visual acuity (inferior or equal to 0.33 decimals/−0.48 logMAR). A suprathreshold strategy (4 dB above the theoretical luminance threshold) was used for the 79 points tested in the central 24 degrees (radius) using a rapid thresholding strategy. When subjects could not execute the test due to a narrow visual field, a central 12 degrees (radius) assessment was performed. Testing was always carried out with appropriate refractive correction. Fixation was monitored in real time with an integrated camera by the operator. The static visual fields of 1 patient (subject 13 in Table 2) were evaluated with Humphrey Field Analyzer (Carl Zeiss Meditec, Germany) for the 10 central degrees due to unavailability of the MonCv3 multifunction perimeter on the day of examination. Visual field extent was then estimated for each eye of each patient in angles of visual degree (diameter). The maximum diameter of visual field was approximately estimated by defining the absolute scotoma as the black region (lower than 10 dB of sensitivity) obtained in the static perimetry sensitivity map (Fig. 3). Visual field mean deficit (in dB) was also obtained.

Frequency Domain Cirrus OCT (software version 5.1.1.6, Carl Zeiss Meditec AG, USA) was performed to measure retinal thickness and retinal nerve fiber layer (RNFL) thickness in all participants, except in 1 patient due to the inaccessibility of the device (subject 23 in Table 2). A macular scan with a resolution of 512 × 128 and an optic disc scan with a resolution of 200 × 200 were acquired.

Age of symptoms onset was self-reported by patients to determine the disease duration (Table 2).

### MRI acquisition

High-resolution brain anatomical images were acquired on a 3 T scanner (Magneton TrioTim, Siemens AG, Germany), using a 12-channel birdcage head coil. One 6-min T_1_-weighted magnetization-prepared rapid acquisition with gradient echo (MPRAGE) sequence was acquired per participant: repetition time (TR) 2.53 s, echo time (TE) 3.42 ms, flip angle (FA) 7°, field of view (FOV) 265 × 256 mm^2^, resulting in 176 slices with 1 × 1 × 1 mm^3^. Brain scans of 2 patients and 2 matched controls were acquired in the same scanner but with different MPRAGE sequence parameters: TR 2.3 s, TE 2.98 ms, FA 9°, FOV 256 × 256 mm^2^, resulting in 160 slices with 1 × 1 × 1 mm^3^. The acquisition time was approximately 10 minutes for this sequence.

### Statistical analysis of demographic and visual data

The statistical analysis was performed with IBM SPSS Statistics (version 23.0, IBM Corporation, USA). Continuous variables were tested for normality, separated by group, using the Kolmogorov-Smirnov test. Differences between the RP and control groups for non-normally distributed variables were tested using the Mann-Whitney *U* test (age, visual acuity, and visual field’s mean deficit), while normally distributed variables were compared using the independent-samples *t*-test (retinal thickness and RNFL thickness). Gender and handedness comparisons between groups were performed using the Pearson’s chi-squared test (χ^2^). Comparisons between the left and right eye within each group were performed using the nonparametric Wilcoxon signed-rank test or the parametric paired-samples *t*-test. Correlations between disease duration and visual field diameter were investigated within the RP group using the non-parametric Spearman test, given the non-normality distribution of the corresponding variables. All *p* values below 0.05 were considered statistically significant.

### VBM data analysis

#### Image processing

T_1_-weighted images were processed using the SPM12 version (Statistical Parametric Mapping, http://www.fil.ion.ucl.ac.uk/), compiled with the MATLAB version R2014a (The MathWorks, Inc., USA).

Firstly, every scan was aligned to the anterior commissure manually. Then, the unified segmentation algorithm^42^ was used to normalize, segment, and modulate the images. All T_1_-weighted images were registered to the Montreal Neurological Institute (MNI) coordinate system by registering the MRI images to the ICBM 152 template. Normalized images were segmented into gray matter (GM), white matter, and cerebrospinal fluid. GM images were modulated to correct for GM volume changes due to nonlinear registration. Additionally, the images were smoothed with a Gaussian kernel with 8 mm of full width at half maximum (FWMH) to ensure the normality of the data. Finally, voxel wise statistical analyses were applied to these processed GM images.

Significant clusters were overlaid in a T1 image of a random healthy subject registered to the MNI space for visualization purposes. This image can be found in the SPM folder. Anatomical labeling was performed in accordance with the work of Tzourio-Mazoyer *et al*.^43^ using the xjview8 toolbox.

### Statistical analysis

GM volume alterations were assessed by comparing RP patients with controls, using the standard general linear model (GLM) implementation in SPM12 for independent two-samples *t*-tests. Statistical parametric maps of two different contrasts (Controls > RP and RP > Controls) were created by applying a significant threshold of *p* < 0.01 with FDR correction for multiple comparisons and a cluster size of 20 voxels. Even though participants were age- and gender-matched (see the Results section), age and gender were included in the model as nuisance covariates. The total intracranial volume (TIV), known to be an important confound in VBM studies^44^, was also included as a nuisance variable. Nonetheless, no significant differences were found between groups for the TIV [independent samples *t*-test *t*_(64)_ = −6.12, *p* = 0.543]. The TIV was calculated in MATLAB using the automated method as previously reported^45^.

To explore possible correlations between GM volume and patient’s visual characteristics (visual field diameter, visual field mean deficit, visual acuity, retinal thickness, and RNFL thickness) as well as disease duration, post-hoc whole brain SPM multiple regression analyses were run. Since no statistical differences were found between the left and right eyes for all visual parameters within the RP group (see the Results section), left- and right-eye clinical parameters were averaged for the subsequent analysis to reduce the number of statistical comparisons. In this way, we excluded the 5 RP patients with missing data from both eyes when running the correlation analyses (subjects 3, 16, 18, 23, and 24 in Table 2). Again, age, gender, and TIV were added as nuisance variables to the regression model, and statistical parametric maps of direct (positive) or inverse (negative) correlation with visual field diameter, visual field mean deficit, visual acuity, retinal thickness, RNFL thickness or disease duration were computed using a *p*-value threshold (<0.001) with a cluster correction of 150 voxels.

## References

[1] Hartong DT, Berson EL, Dryja TP (2006). Retinitis pigmentosa. Lancet.

[2] Hamel C (2006). Retinitis pigmentosa. Orphanet journal of rare diseases.

[3] Cottet S, Schorderet DF (2009). Mechanisms of apoptosis in retinitis pigmentosa. Current molecular medicine.

[4] Herse P (2005). Retinitis pigmentosa: visual function and multidisciplinary management. Clinical & experimental optometry: journal of the Australian Optometrical Association.

[5] Andrea Mechelli, C. J. P., Karl J. F, John Ashburner Voxel-Based Morphometry of the Human Brain: Methods andApplications. *Current Medical Imaging Reviews* **1**, 00–00 (2005).

[6] Ashburner J, Friston KJ (2000). Voxel-based morphometry–the methods. NeuroImage.

[7] Whitwell JL (2009). Voxel-based morphometry: an automated technique for assessing structural changes in the brain. The Journal of neuroscience: the official journal of the Society for Neuroscience.

[8] Boucard CC (2009). Changes in cortical grey matter density associated with long-standing retinal visual field defects. Brain: a journal of neurology.

[9] Hernowo AT, Boucard CC, Jansonius NM, Hooymans JM, Cornelissen FW (2011). Automated morphometry of the visual pathway in primary open-angle glaucoma. Investigative ophthalmology & visual science.

[10] Barcella V (2010). Evidence for retrochiasmatic tissue loss in Leber’s hereditary optic neuropathy. Human brain mapping.

[11] Plank T (2011). Gray matter alterations in visual cortex of patients with loss of central vision due to hereditary retinal dystrophies. NeuroImage.

[12] Li C (2012). Voxel-based morphometry of the visual-related cortex in primary open angle glaucoma. Current eye research.

[13] Zikou AK (2012). Voxel-based morphometry and diffusion tensor imaging of the optic pathway in primary open-angle glaucoma: a preliminary study. AJNR. American journal of neuroradiology.

[14] Chen WW (2013). Structural brain abnormalities in patients with primary open-angle glaucoma: a study with 3T MR imaging. Investigative ophthalmology & visual science.

[15] Williams AL (2013). Evidence for widespread structural brain changes in glaucoma: a preliminary voxel-based MRI study. Investigative ophthalmology & visual science.

[16] Park HJ (2009). Morphological alterations in the congenital blind based on the analysis of cortical thickness and surface area. NeuroImage.

[17] Jiang J (2009). Thick visual cortex in the early blind. The Journal of neuroscience: the official journal of the Society for Neuroscience.

[18] Voss P, Pike BG, Zatorre RJ (2014). Evidence for both compensatory plastic and disuse atrophy-related neuroanatomical changes in the blind. Brain: a journal of neurology.

[19] Hernowo AT (2014). Morphometric analyses of the visual pathways in macular degeneration. Cortex; a journal devoted to the study of the nervous system and behavior.

[20] Olivo G (2015). Cerebral Involvement in Stargardt’s Disease: A VBM and TBSS Study. Investigative ophthalmology & visual science.

[21] Wang J (2016). Structural brain alterations in primary open angle glaucoma: a 3T MRI study. Scientific reports.

[22] Kitajima M (1997). MR changes in the calcarine area resulting from retinal degeneration. AJNR. American journal of neuroradiology.

[23] Prins D, Hanekamp S, Cornelissen FW (2015). Structural brain MRI studies in eye diseases: are they clinically relevant? A review of current findings. Acta ophthalmologica.

[24] Wandell BA, Brewer AA, Dougherty RF (2005). Visual field map clusters in human cortex. Philosophical transactions of the Royal Society of London. Series B, Biological sciences.

[25] Wandell BA, Dumoulin SO, Brewer AA (2007). Visual field maps in human cortex. Neuron.

[26] Wandell BA, Winawer J (2011). Imaging retinotopic maps in the human brain. Vision research.

[27] Triolo G (2013). Spectral domain optical coherence tomography findings in patients with retinitis pigmentosa. Ophthalmic research.

[28] Battaglia, P. M. *et al*. Correlation of SD-OCT findings and visual function in patients with retinitis pigmentosa. *Graefe’s Archive for Clinical and Experimental Ophthalmology*, 1–5, doi:10.1007/s00417-015-3185-x (2015). 10.1007/s00417-015-3185-x 26472300

[29] Hood DC (2009). Thickness of receptor and post-receptor retinal layers in patients with retinitis pigmentosa measured with frequency-domain optical coherence tomography. Investigative ophthalmology & visual science.

[30] Oishi A (2013). Longitudinal analysis of the peripapillary retinal nerve fiber layer thinning in patients with retinitis pigmentosa. Eye (London, England).

[31] Anastasakis A, Genead MA, McAnany JJ, Fishman GA (2012). Evaluation of retinal nerve fiber layer thickness in patients with retinitis pigmentosa using spectral-domain optical coherence tomography. Retina (Philadelphia, Pa.).

[32] Garaci FG (2009). Optic Nerve and Optic Radiation Neurodegeneration in Patients with Glaucoma: *In Vivo* Analysis with 3-T Diffusion-Tensor MR Imaging. Radiology.

[33] Hernowo AT, Boucard CC, Jansonius NM, Hooymans JMM, Cornelissen FW (2011). Automated Morphometry of the Visual Pathway in Primary Open-Angle Glaucoma. Investigative ophthalmology & visual science.

[34] Zhang, Y. *et al*. Reduced Field-of-View Diffusion Tensor Imaging of the Optic Nerve in Retinitis Pigmentosa at 3T. *AJNR*. *American journal of neuroradiology*, doi:10.3174/ajnr.A4767 (2016). 10.3174/ajnr.A4767 PMC7960274 27056427

[35] Ohno N (2015). Alteration of the optic radiations using diffusion-tensor MRI in patients with retinitis pigmentosa. British Journal of Ophthalmology.

[36] Ferreira, S. *et al*. Primary visual cortical remapping in patients with inherited peripheral retinal degeneration. *NeuroImage: Clinical* **13**, 428–438, doi:10.1016/j.nicl.2016.12.013 (2017). 10.1016/j.nicl.2016.12.013 PMC5233796 28116235

[37] Shintani K, Shechtman DL, Gurwood AS (2009). Review and update: current treatment trends for patients with retinitis pigmentosa. Optometry (St. Louis, Mo.).

[38] Fernandez E (2005). Development of a cortical visual neuroprosthesis for the blind: the relevance of neuroplasticity. Journal of neural engineering.

[39] Schoth F, Burgel U, Dorsch R, Reinges MH, Krings T (2006). Diffusion tensor imaging in acquired blind humans. Neuroscience letters.

[40] Brown HD, Woodall RL, Kitching RE, Baseler HA, Morland AB (2016). Using magnetic resonance imaging to assess visual deficits: a review. Ophthalmic & physiological optics: the journal of the British College of Ophthalmic Opticians (Optometrists).

[41] Cunningham SI (2015). Feasibility of Structural and Functional MRI Acquisition with Unpowered Implants in Argus II Retinal Prosthesis Patients: A Case Study. Translational vision science & technology.

[42] Ashburner J, Friston KJ (2005). Unified segmentation. NeuroImage.

[43] Tzourio-Mazoyer N (2002). Automated anatomical labeling of activations in SPM using a macroscopic anatomical parcellation of the MNI MRI single-subject brain. NeuroImage.

[44] Barnes J (2010). Head size, age and gender adjustment in MRI studies: a necessary nuisance?. NeuroImage.

[45] Pengas G, Pereira JM, Williams GB, Nestor PJ (2009). Comparative reliability of total intracranial volume estimation methods and the influence of atrophy in a longitudinal semantic dementia cohort. Journal of neuroimaging: official journal of the American Society of Neuroimaging.

